# Induced electron radiation effect on the performance of inter-satellite optical wireless communication

**DOI:** 10.1371/journal.pone.0259649

**Published:** 2021-12-31

**Authors:** Abdouraouf Said Youssouf, Nurul Fadzlin Hasbullah, Norazlina Saidin, Mohamed Hadi Habaebi, Rajendran Parthiban, Muhammad Rawi Bin Mohamed Zin, Elfatih A. A. Elsheikh, F. M. Suliman

**Affiliations:** 1 Department of Electrical and Computer Engineering, International Islamic University Malaysia, Kuala Lumpur, Malaysia; 2 Department of Electrical and Computer Engineering, School of Engineering, Monash University Malaysia, Subang Jaya, Malaysia; 3 Radiation Processing Technology Division, Nuclear Malaysia, Bangi, Malaysia; 4 Department of Electrical Engineering, College of Engineering, King Khalid University, Abha, Saudi Arabia; Nanchang University, CHINA

## Abstract

This paper provides the details of a study on the effects of electron radiation on the Performance of Inters-satellite Optical Wireless Communication (IsOWC). Academia and industry focus on solutions that can improve performance and reduce the cost of IsWOC systems. Spacecraft, space stations, satellites, and astronauts are exposed to an increased level of radiation when in space, so it is essential to evaluate the risks and performance effects associated with extended radiation exposures in missions and space travel in general. This investigation focuses on LEO, especially in the near-equatorial radiation environment. Radiation experiments supported with simulations have made it possible to obtain and evaluate the electron radiation impact on optoelectronics at the device level and system level performances. The electron radiation has induced a system degradation of 70%. This result demonstrates the importance of such an investigation to predict and take necessary and suitable reliable quality service for future space missions.

## Introduction

Recently, some measures have been launched to improve the IsOWC networks to ensure dependability, flexibility, and a large and efficient broadcast. In addition, to support the internet of things through the 5G initiative by processing a vast volume of data created by IoT devices and providing connectivity to billions of devices with varying levels of quality of service (QoS) [1, 2]. Furthermore, with the concept of the implementation of the space-aerial-terrestrial integrated 5G network (SATIN) and the rapid expansion of ubiquitous mobile services, next-generation wireless communication systems, such as beyond 5G (B5G) and 6G systems, will face formidable challenges posed by the need for a large number of concurrent services with extremely high connection density [3, 4].

With the advantages of higher data transmission rates, lower payload power requirements, and smaller terminal size and weight, inter-satellite wireless optical communication (IsOWC) systems have been extensively researched in all aspects to enhance state of the art, such as increasing the speed of transmission by incorporating different wavelengths and modulation techniques [5], or increasing the bandwidth and reduce the cost [6], where the findings suggest that IsOWC could be one of the best alternative ways to meet the growing demands of future high-speed transmission with massive data in space [7–10]. Furthermore, IsWOC could be flexible to accommodate the key features of the future 6G communications, where high security, secrecy and privacy suggested to be the main concerns, besides the new communication scenarios in future network which encompass holographic calls, flying networks, teleoperated driving and the tactile internet and many others [11].

The outer space is known to be dense with high-energy particles such as protons, electrons, neutrons, and heavy ions. When these high-energy particles collide with optoelectronic devices, they cause irreversible damage to the devices’ materials, resulting in substantial performance decline and even malfunction [12, 13]. In 2010, Malaysia launched Razak Sat 1, a satellite equipped with a high-resolution camera for land management, resource development, and forestry, into Near Equatorial Low Earth Orbit. Unfortunately, after a year, the satellite lost contact with the earth’s base station. Radiation is thought to have harmed it, affecting the communication capabilities of its equipment, as described in [14].

Some commercial off-the-shelf (COTS) optoelectronics devices are used in some transmitter and receiver modules of the communication systems, such as in CubeSats [15–18]. Previous research works have been investigated the performance of some optoelectronics components under radiation deployed in satellite space missions. They show that significant parts of satellite communication systems could be affected by the characteristics of the radiation environment [19, 20]. Few have been reported the degradation of the performance at the system level. Therefore, it is of considerable importance to investigate the effect of radiation on the performance of the optoelectronic components, namely the lasers and photodiodes implemented in IsOWC systems under radiation, and simulate their induced performance degradation at the system level.

The rest of this paper is organized as follows. Section II briefly explains the effects of radiation on semiconductors, inter-satellite Optical wireless Communication, and performance evaluation. In Section III, we demonstrate the methodology of the electron irradiation experiment of the optoelectronic devices, namely the laser and the photodiode. Next, the discussion of the results of the device’s degradation and system performance under electron radiation is simulated in Section IV. The conclusions are drawn in Section VI.

## Theory

Physics and device simulations study have always been viable alternatives for space exploration as it is difficult, hazardous, and costly to emulate high-energy space particles in the lab. The study of radiation effects and radiation hard electronics related to space missions is still in its infancy. Given the possibilities, we will practically radiate the transmitters and photodetectors with electron radiation. Pre-determined parameters will be measured before and after radiation to quantify the radiation effects on the optical satellite communication system. Then, an optical inter-satellite communication link will be designed using OptiSystem. The magnitude degradation of the devices under test will be fed to the system, and the link’s performance will be simulated using Optisystem software.

### Effect of radiation on semiconductors

Optoelectronic semiconductor devices operated in a radiation environment such as space are subject to degradation due to radiation. The high-energy charged particles and heavy ions would possibly impose ignored influence on the laser communication equipment and sometimes result in their malfunctions. According to the existing research results [21–24], laser source and photoelectric detector equipment were paid much attention. However, few research studies under space radiation environments were rarely researched; few are available lately.

It has been revealed that the main damage mechanisms induced by space radiation to semiconductor materials are ionization damage, surface damage, and displacement damage. The ionization damage is generally caused by γ radiation, and the ionizing radiation makes superfluous electron-hole pairs leading to the conduction increase [25]. The surface damage is the ionization process of the surface oxidation layer caused by ionizing radiation, and it introduces a positive electric charge and surface status. All three damage mechanisms could cause the decrease of the output power d of the laser source and the shift of central wavelength [26–31]. On the other hand, it will lead to the decrease of the photodetector’s responsivity and the increase of dark current [32, 33]. As a result, the BER performance of the communication system will be affected [19, 25, 34, 35].

### Inter-satellite optical wireless communication

With the need for satellite to satellite links, inter-satellite communication is mainly employed to provide global coverage. Intra-orbital and inter-orbital linkages are the two forms of ISL. Intra-orbital linkages link satellites in the same orbit together, whereas inter-orbital links link satellites in different orbits together [36]. Because of their smaller footprints and the necessity for worldwide coverage, LEO and MEO satellite networks are more common for inter-satellite communication [37].

Apart from LEO and MEO satellite networks, they can also be used for communication between the GEO satellite, the LEO satellite, and the ground. With the rising demand for information at higher data rates, optical communication-based ISL has the advantage of providing high-speed communication with the data rate of the order of gigabits per second (Gbps) [38–40].

The IsOWC system’s modulation techniques were critical in achieving a high-quality transmission. The phase-shift keying (PSK) approach has been proven to be a very good modulation technique for space optical communication systems, as adaptive thresholding methods are easily overcome [41]. As a result, selecting a modulation technique is an important decision to make throughout the system’s design. The typical modulation scheme that has been employed for onboard demonstrations is homodyne BPSK. In the communication link between TerraSAR-X and the Near-Field Infrared (NFIRE) experiment, the homodyne BPSK method is used. As a result, homodyne BPSK will be one of the essential modulation methods for future optical inter-satellite communication missions [42]. On the other hand, considering the threat of space ionizing radiation’s impact on the on-orbit performance of the communication systems, investigations of the performance of the IsOWC system with this type of modulation scheme should be mainly studied. In previous investigations, as reported in [12, 43]gamma radiation impacts on 1550 nm OOK, DPSK, and homodyne BPSK based optical inter-satellite communication systems were studied on-orbit performance degradation of 1550 nm BPSK based systems was predicted.

This research aimed to offer a study that included the 980 nm IsOWC under electron radiation to cover a wide range of wavelengths and types of radiation sources, in line with previous research. To the best of our knowledge, this study may be one of the first to look at the effects of induced electron radiation on the IsOWC system’s performance.

Thanks to OptiSystem software, a typical IsOWC with 980 nm of wavelength is designed as reported in Table 1. The system performance degradation implementing homodyne BPSK based inter-satellite system is simulated based on the analytical analyses and experimental results.

**Table 1:** Information of devices used for the experiment.

No	Mode	Materials	Wavelength	Type of device
1	L980P010	AlGaAs	980 nm	laser
2	FCI-InGaAs-300	InGaAs	900nm to 1700nm	Photodiode

### Analytical characterization of the system performance

**Fig 1:**
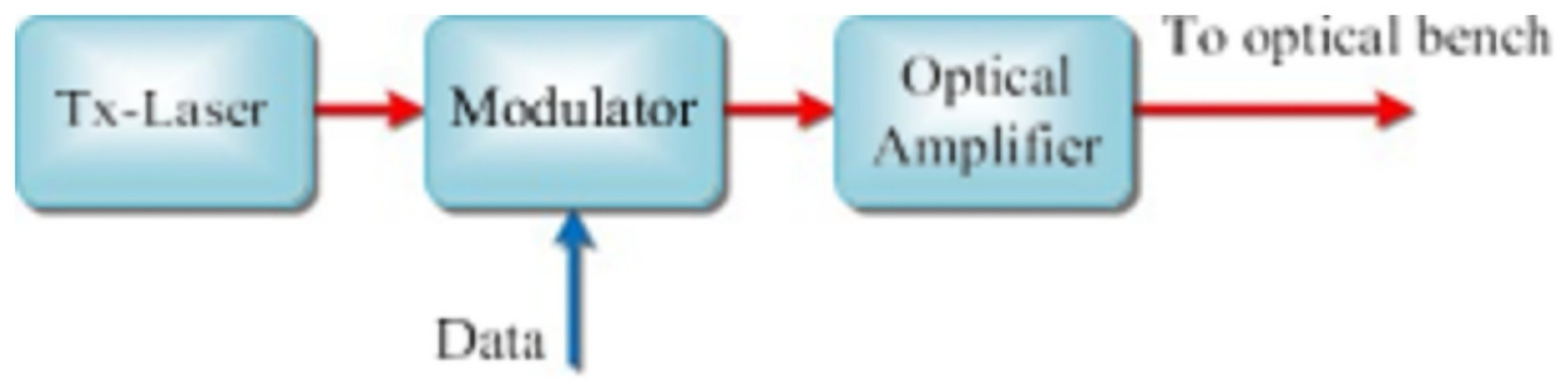
Transmitter structure.

The communication transmitters (Tx) and receiver (Rx) designs are based on previous similar investigations [12, 20, 35, 44]. As is shown in Fig 1, Tx consists of a 980 nm CW Tx-Laser, a LiNbO3 amplitude/phase modulator, and an optical amplifier.

Tx output signal power is given:

$$S_{t}=P_{LD}T_{m}G_{t}$$ (1)

where *P*_LD_ is the output power of Tx laser, *T*m is the optical transmission of modulator, and *G*t is the gain of EDFA. Additionally, when sending code "0", we assume that no optical signal power is transmitted. For BPSK based systems with phase modulation, *S*_*t*_ = *P*_*LD*_*T*_*m*_*G*_*t*_. The optical signal from Tx is transmitted into the free space channel and then received by the receiver (Rx) on the destination satellite. The expression of received signal power can be given as:

$$P_{r}=S_{r}+N_{r}=(S_{t}+Nt)L_{link}$$ (2)

where *S*r is the received signal power, *S*_*r*_ = *S*_*t*_*L*_*Link*_, *N*_*r*_ is the received noise power, *N*_*r*_ = *N*_*t*_*L*_*LINk*_, *N*_*t*_ is the transmitted noise power, *L*_*Link*_ is the loss of optical link, including geometrical loss, coupling loss, transmitter and receiver efficiency loss, and ATP loss.

**Fig 2:**
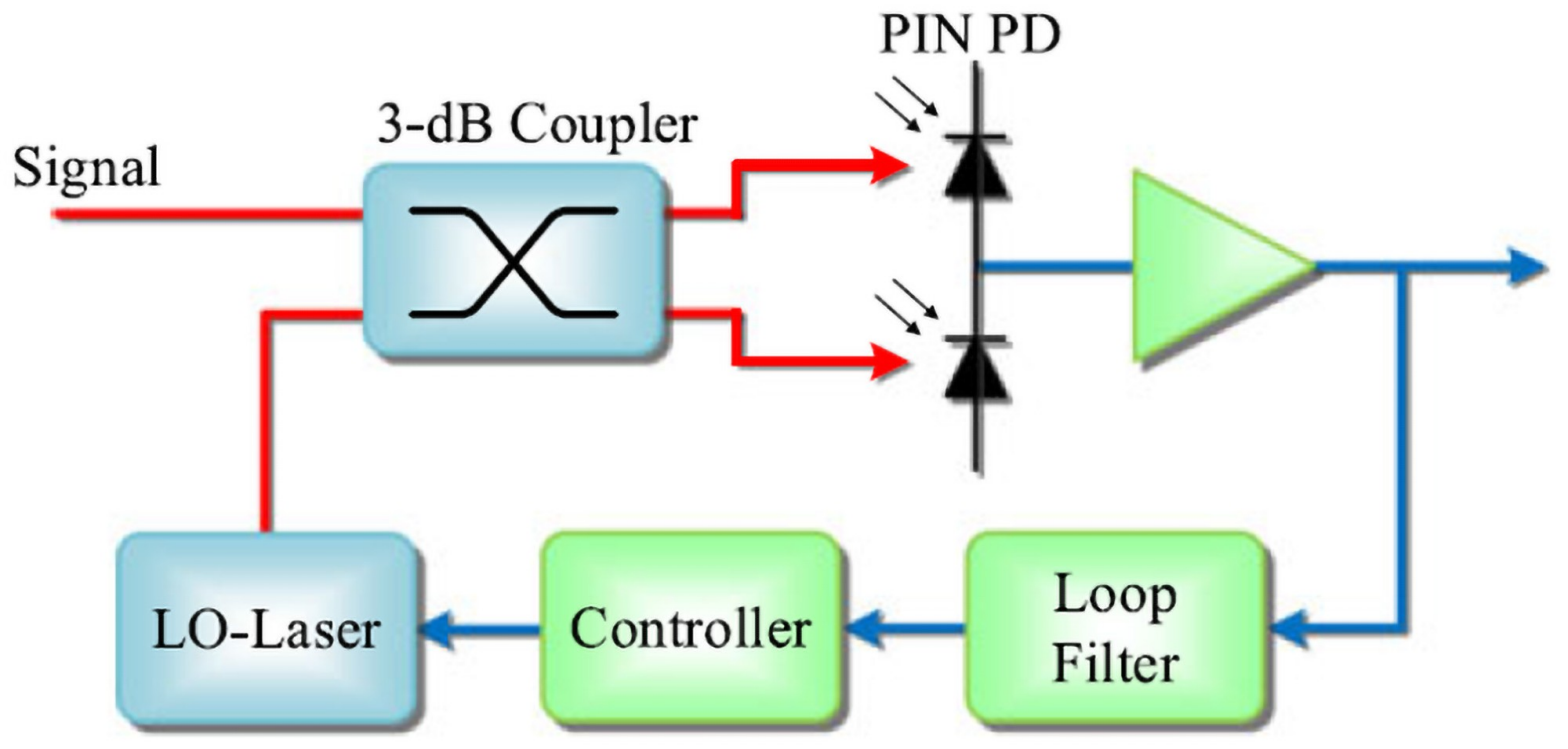
Diagram of the structure of the BPSK receiver [12].

The structure of homodyne BPSK based Rx is shown in Fig 2. The received light beam and local laser beam are guided into a 3-dB coupler, and the outputs of the coupler are passed on to photodiodes. By subtracting the photocurrent of the outputs of two photodiodes, we can obtain the signal current of the BPSK based system.

$$i_{s}(t)=2R\sqrt{S_{r}P_{LO}}cos(\phi (t))$$ (3)

Where *P*LO is the optical power of the local laser, since this BPSK homodyne receiver does not have an optical preamplifier, ASE noise can be ignored while calculating noise power. Equation of noise power is given by:

$$\sigma^{2}=qBR(P_{r}+P_{LO})+2qBI_{D}+4k_{B}TB/R_{L}$$ (4)

The Q factor of homodyne BPSK based system correspondingly becomes:

$$Q_{BPSK}=\frac{2R\sqrt{S_{r}P_{LO}}}{\sqrt{qB(R(P_{r}+P_{LO})+I_{D})+4k_{B}TB/R_{L}}}$$ (5)

Hence, expression of BER can be given by:

$$BER_{BPSK}=\frac{1}{2}erfc(\frac{2R\sqrt{S_{r}P_{LO}}}{\sqrt{2qB(R(P_{r}+P_{LO})+I_{D})+8k_{B}TB/R_{L}}})$$ (6)

## Methodology

### Experiment setup

The experimental setup of the irradiation process will be encompassing two stages. Firstly, the optoelectronics devices’ irradiation process, namely the lasers and photodiodes, is done with the EPS– 3000 electron beam machine facility in Nuclear Malaysia, which could provide up to 3 MeV of electron radiation. For considering the worst-case scenario and with reference to Suparta [14] The samples were irradiated with 1.5 MeV electrons of energy with five iterations of radiation exposure of electron-equivalent doses ranging from 50 kGy to 200 kGy, at the Malaysian Nuclear Agency.

### Parameter’s characterization

Before and after the irradiation process, the electrical and optical characterization of all samples was carried out in the photonics and optoelectronics labs at University Malaya and IIUM. The selected optoelectronics components are from the transmitters and the receivers’ subsystems, namely the laser and photodetectors. The following laser has been selected for the experiment the L980P010**–**5.6 mm, TO-18, 980 nm laser diode. For the photodiodes, the FCI-InGaAs-300 is selected. Table 1 shows the details of samples chosen for laser and photodiodes. All devices are commercial on the shelf (COTS), where they are chosen based on their manufacturing materials and wavelengths in concordance with the specifications of the IsOWC systems applications.

### Simulation of the system performance with OptiSystem

**Fig 3:**
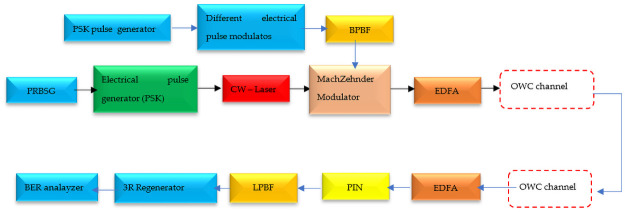
The layout of the simulation of the inter-satellite optical wireless communication in OptiSystem.

The simulation of the IsOWC system performance is carried out using the OptiSystem software version 17. The layout is reported in Fig 3. A pseudorandom binary sequence generator (PRBSG) is used to produce several sequence bits ("0s and 1s") based on the information length, which is subsequently directed to various electrical pulse modulators and generators. Electrical pulse generators control pulse repetition rate and pulse width, and electrical pulse modulators such as PAM, DPSK, or BPSK, as in our case, are used to convert a narrowband analog signal to a bit stream across a digital transmission system using a band Pass Bessel filter (BPBF). The Mach Zehnder modulator receives the output signal from electrical pulse generators as well as the output signal from the modulator (MZM) [45].

At a wavelength of 980 nm and a coverage distance of 1000 km, the output signal form (MZM) is directed to the EDFA and subsequently to the optical wireless communication (OWC) channel. In this investigation, for accuracy, the signal has not been amplified for the purpose of demonstrating the radiation effect on the laser and PIN photodiode; instead, it is directly fed to the Bessel filter and then routed to the PIN photodetector before being reshaped by the 3R regenerator and displayed on the BER analyzer.

The simulated results will be obtained by inserting experimental data of devices into the design IsOWC communication link established utilizing OptiSystem software with key parameters as reported in Table 2, and according to space satellite missions at LEO [6].

**Table 2:** Parameters for simulation.

Parameters	Values
WOC type	Line-of-sight
Bit-rate, Gbps	10 Gbps
Modulation	BPSK
Wavelengths, nm	980 nm
Link range	1000 km
Output power, dBm	30
Sequence length	128
Samples per bit	64
Line width of laser, MHz	0.1
Dark current, nA	10
Responsivity A/W	1
Tx aperture, mm	150
Rx aperture, mm	150

## Results and discussion

### Characterization of the laser and photodiode before and after electron radiation

**Fig 4:**
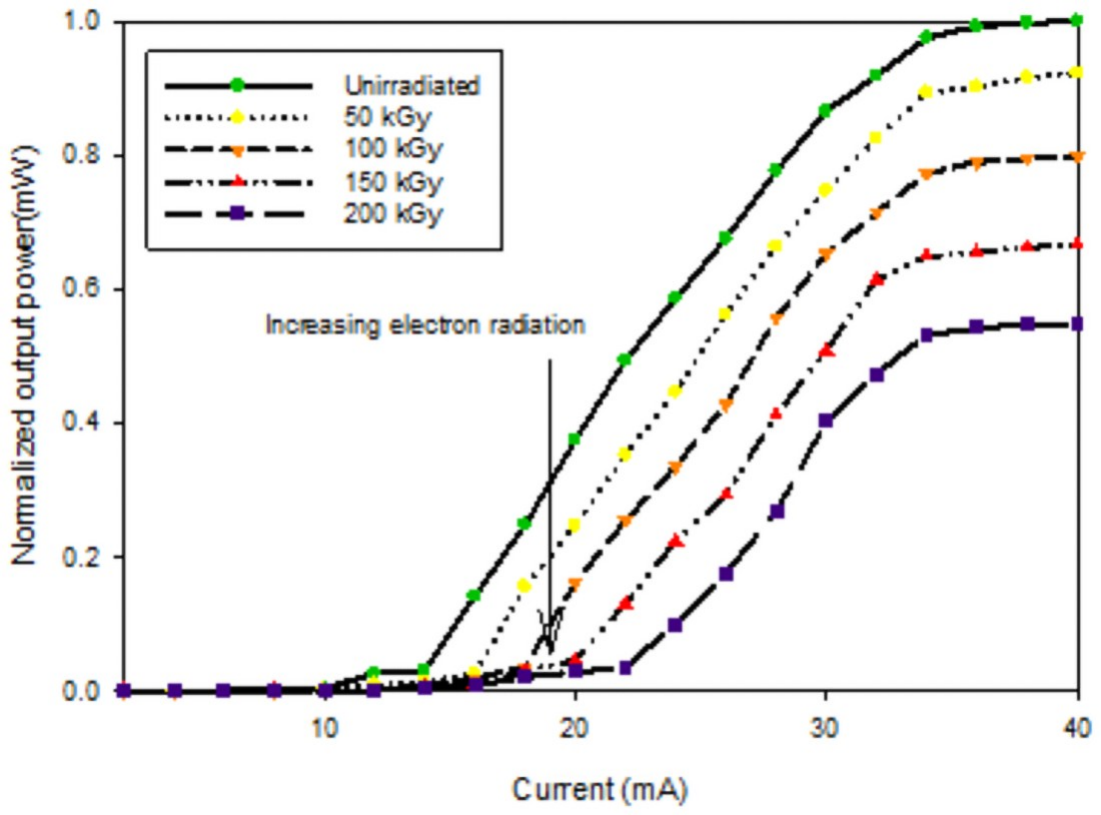
Degradation of the 980 nm laser diode: L-I characteristics under electron radiation.

The measurement of the devices’ performance parameters before and after irradiation is reported and discussed in the following section. The laser output power has been used as the main parameter to put into evidence and the dark current for the photodiode. Fig 4 represents the normalized output power versus current characterization. Before irradiation, the output power curve corresponds to the specifications reported in the datasheet.

**Fig 5:**
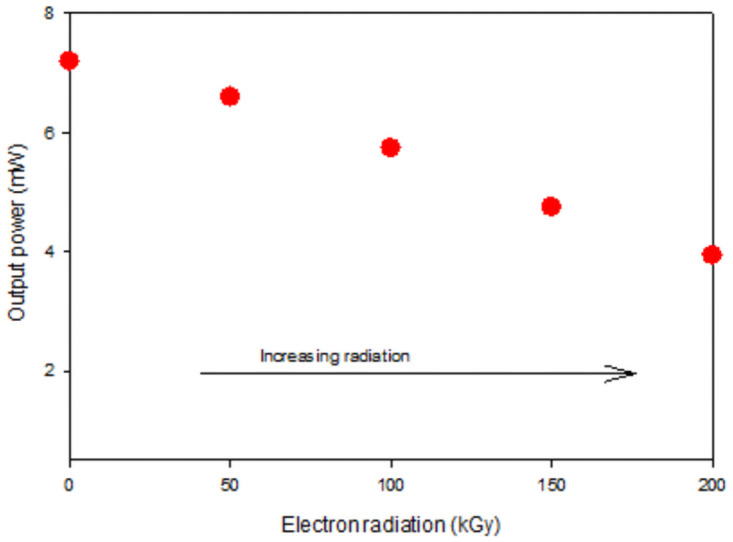
Degradation of the 980 nm laser diode: Radiation-induced optical power loss at 30 mA.

The threshold current and the slope efficiency can be investigated besides the output power with the increase of the electron dose. Before irradiation, the threshold current is observed at 10 mA, and the output power was 7.2 mW. After the first radiation dose, the threshold current has been shifted by more than 6 mA, while the output power dropped by less than 1 mW. The same trend can be observed in the subsequent radiation doses illustrated in Fig 5, which displays the output power versus radiation doses.

After 100 kGy, the threshold current shifted significantly more than 3 mA and the output power, decreasing about 1mW. However, after the last dose of 200 kGy of electron irradiation, the threshold current shifted by more than 6 mW compared to the unirradiated threshold current, while the output power dropped from 7 mW to 4 mW approximately which equivalent to a decrease of more than 40% from the total output power.

The results conform with the previous investigations reported in [16], where the output was degraded, the threshold current shifted with the increase of radiation dose. It has been stated in [17] that the evolution of threshold current with irradiation and the slope efficiency at higher irradiation dose. It has been reported in [18] that irradiation induces non-radiative recombination centers, which decreases the carrier lifetime. This leads to an increase in threshold current but unchanged slope efficiency.

**Fig 6:**
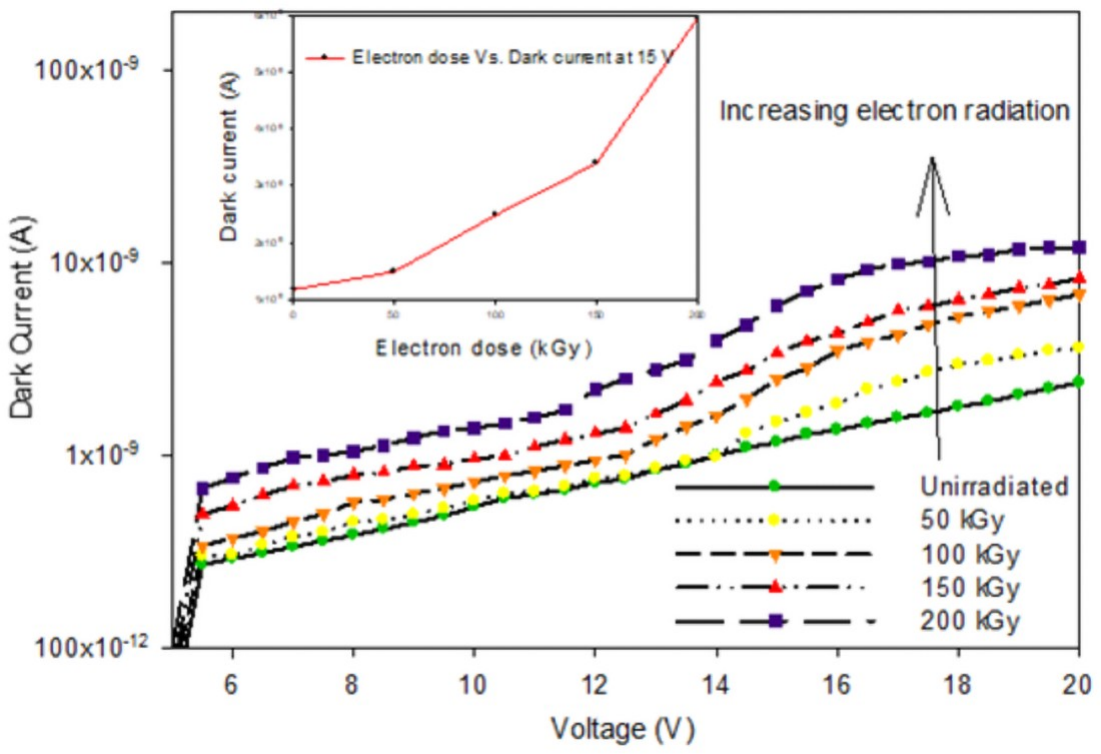
Electron irradiation of dark current versus voltage.

The dark current has been used as the parameter to investigate the electron radiation effect on the InGaAs Photodiode for the photodiodes. Fig 6 shows the characterization of the dark current. By analyzing closely, before radiation, the dark current was 1.35 nA, which is typical to a photodiode operating at this particular wavelength.

The inset shows the dark current versus radiation doses, and it can be seen that the dark current keeps increasing with more electron radiation exposure. After 200 kGy of exposure, the dark current increases to 40 nA, at 20 V. This finding follows previous discovery in [20], that electron irradiation causes defects in the intrinsic epilayer of PIN diodes in the formation of deep acceptor levels in the bandgap.

### Communication performance degradation

The simulated results will be obtained by inserting experimental data of devices into communication links established utilizing OptiSystem software with key parameters as reported in Table 2, and according to space satellite missions at LEO [6]. The electron irradiation impact on inter-satellite optical communication terminals has been analyzed in the previous section, and by combining the effects on Tx and Rx, system performance under electron radiation can be subsequently simulated. The normalized Q factor and BER for the homodyne BPSK based system, as a function of both Tx and Rx dose, are obtained.

**Fig 7:**
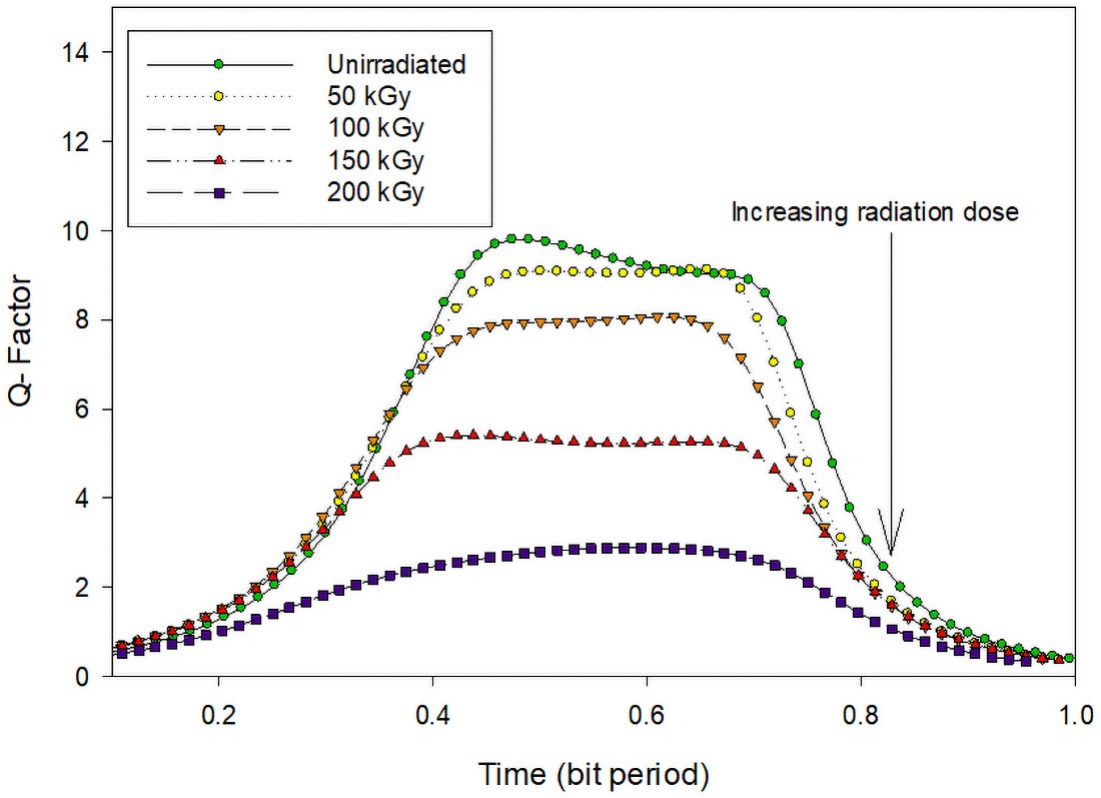
Electron radiation effect on Tx, with 980 nm laser.

**Fig 8:**
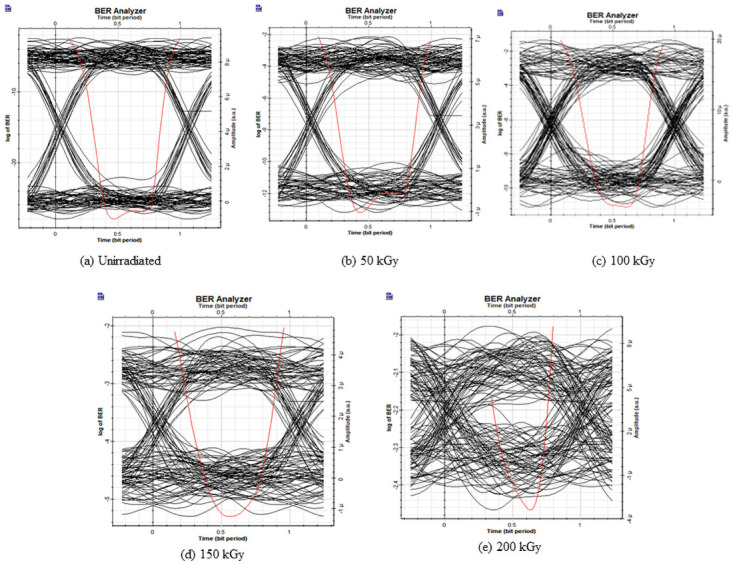
Eye diagram for the IsOWC link, 980 nm with radiated Tx.

To investigate the system performance at 980 nm, Figs 7 and 8 reports the Q factor, BER, and the eye diagrams before and after electron irradiation. Before irradiation, the Q factor of the system was 10 with a BER of 4 × 10^−26^.

Fig 7 represents the Q factor of the inter-satellite optical communication links performance that implements the 980 nm laser in the transmitter under different irradiation doses. Before irradiation, the Q factor of the system was 26 with a BER of 1.5 × 10^−26^.

After the irradiation process with different doses, the Q factor decreases. At 200 kGy the system performance deteriorates badly where the BER increases up to 1.3 × 10^−4^. The effect of the electron radiation on the system performance can be seen through the eye diagrams in Fig 8.

**Fig 9:**
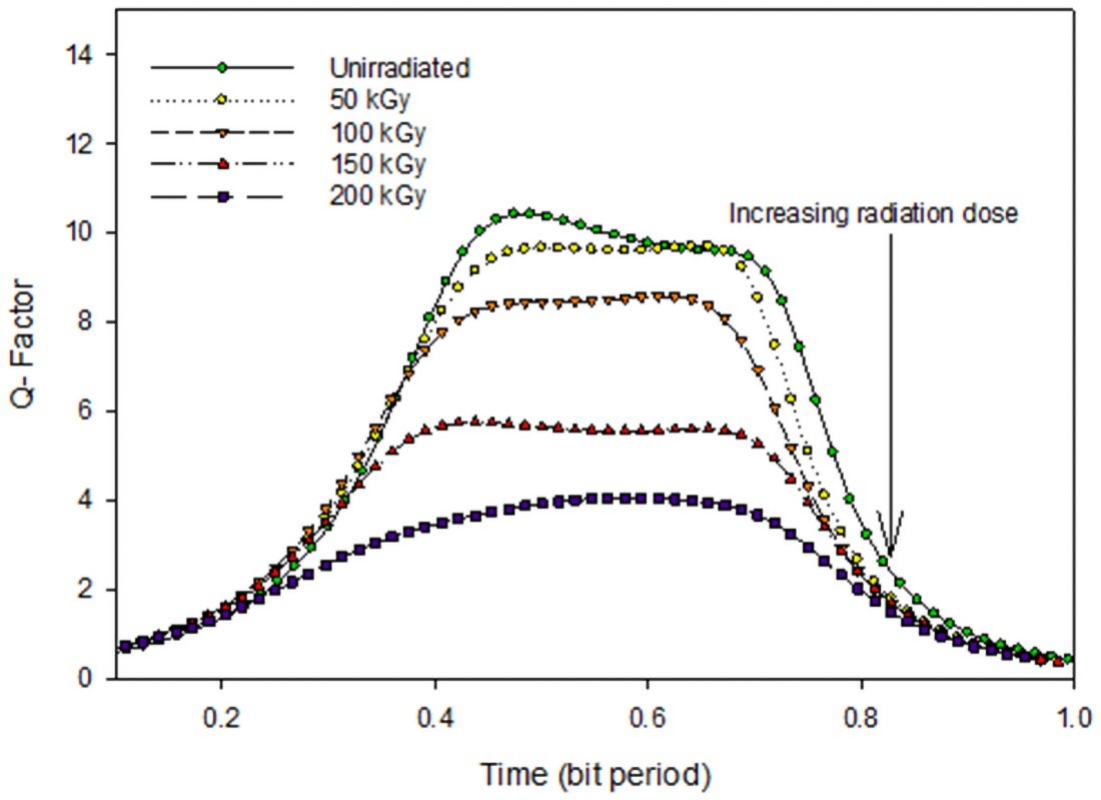
Electron radiation effect on Rx, with 980 nm laser.

**Fig 10:**
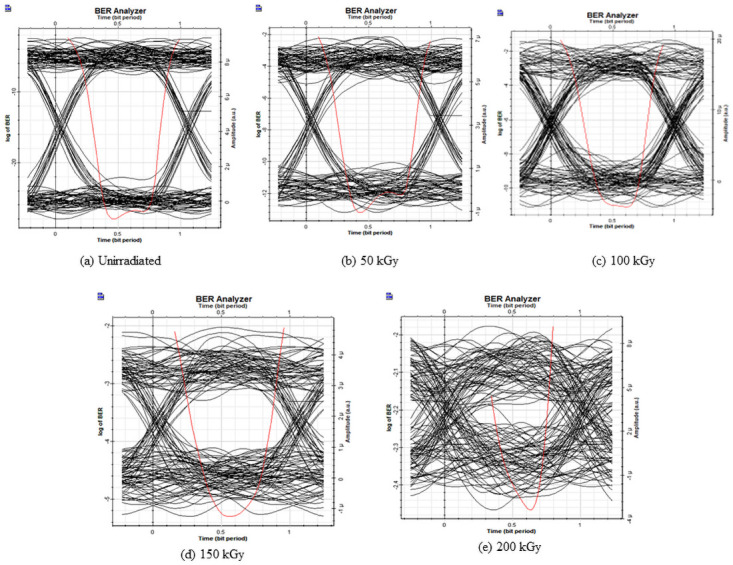
Eye diagram for the IsOWC link, RX under electron radiation 980 nm.

The effect of the electron radiation effect on photodiodes is put into evidence in the following section. Fig 9 represents the performance of the system considering the degradation of the photodiode under electron radiation of 980 nm. Before irradiation, similarly, for the Tx, the Q factor was around 26 with a BER of 1.5 × 10^−26^. After 50 kGy of electron radiation exposure to the photodiodes, the system performance degraded where the Q factor decreases to 9 with 1.5 × 10^−16^ and after 200 kGy of electron radiation dose, the Q factor decreases to 4 with a BER of 2.8 × 10^−5^ approximately. Fig 10 further illustrates the electron radiation effects on the photodiodes with the eye diagrams.

### Effect on overall system on-orbit performance

**Fig 11:**
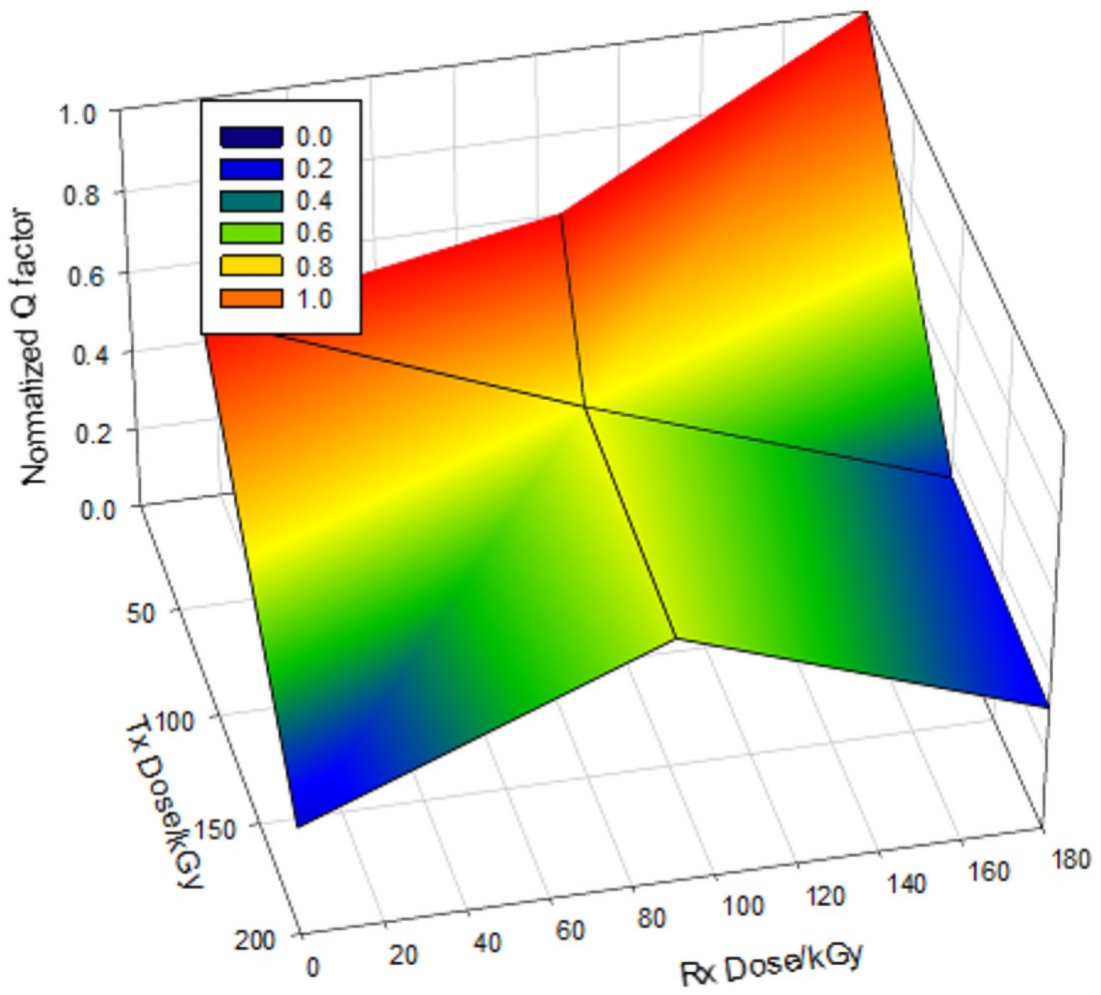
Normalized Q factor versus electron radiation dose on Tx and Rx–BPSK.

**Fig 12:**
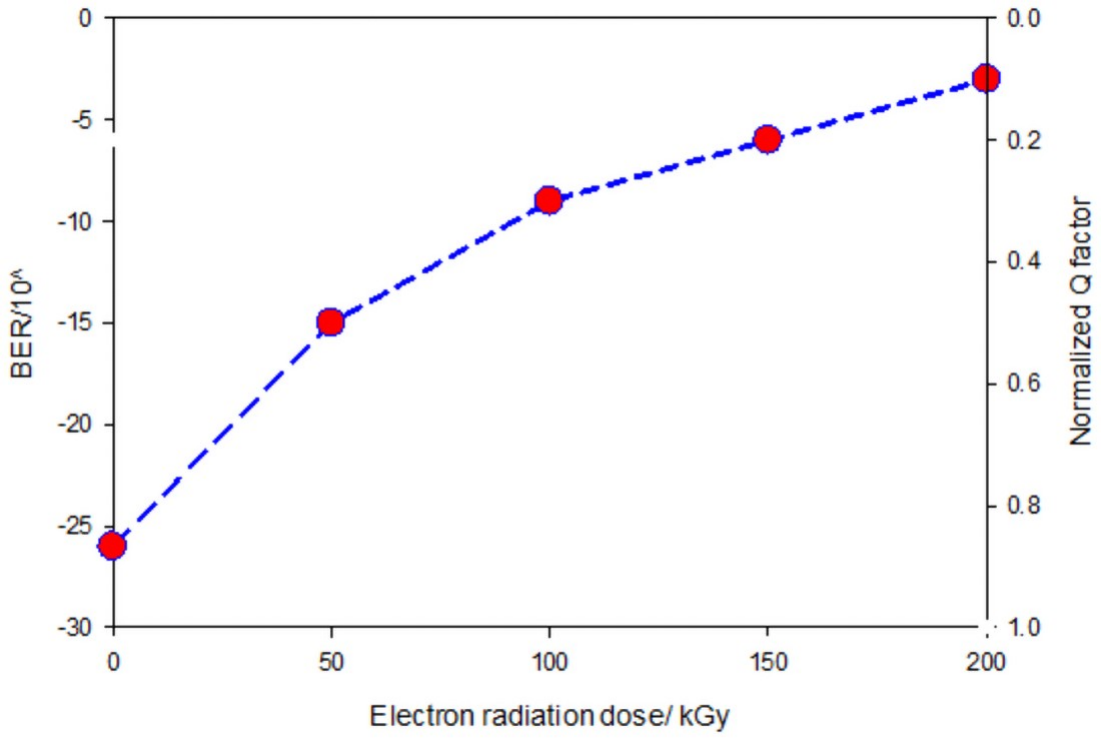
BER and Q factor versus electron radiation dose on Tx and Rx -BPSK.

The degradation of the normalized Q factor of the homodyne BPSK based system, as a function of both Tx and Rx dose, is obtained as reported in Fig 11. It shows the normalized Q factor versus the electron radiation dose model. The normalized Q factor is one before irradiation, as can be shown. The Q factor drops by 20% when the radiation effect of 50 kGy is combined on the Tx and Rx. The degradation of the system deteriorating as the electron radiation dose was increasing. After 200 kGy of electron irradiation, the Q factor decreases by more than 80%, an indicator of system failure. This can be further illustrated in Fig 12, where the BER is increased to 1 × 10^−3^, which is below the threshold of the accepted range of BER.

It can be observed in Table 3 that both radiations have resulted in system performance degradation, which is a comparison between the findings of this study and those of other comparable investigations. It should be noted that in this study, the EDFA (amplifier) was not used during the simulation process. Simulation of the combined radiation effect on a laser and a photodiode has been carried out without the use of the EDFA’s amplifier. Beside the high bit rate, this could explain why the BER for this experiment increased when compared to Liu et al. [17].

**Table 3:** Comparative analysis of the present investigation with previous studies.

Parameters	Liu et al. [20]	Present investigation
Modulation	BPSK	BPSK
Type of radiation	Gamma	Electron
Distance (km)	20000	1000
Data rate (Gbps)	1.0	10
Wavelength, nm	1550	980
Pre-irradiation BER	2.56 × 10^−33^	1.5 × 10^−26^
Post- irradiation BER	1.0 × 10^−3^	1.0 × 10^−3^

## Conclusions

The irradiation experiment of the selected optoelectronics components, namely the laser and the photodiodes for the electron radiation, has been carried out successfully. Electron radiation has made a significant impact on devices. The characterization of the devices demonstrates the magnitude of the radiation effect, where the laser diodes threshold current density increased, and the output power decreased with the increase of irradiation doses by more than 40%. The decrease of quantum efficiency translates this degradation by creating non-radiative recombination centers that compete with radiative recombination sites. For the photodiode, the dark current increases by 40 nA, which led the responsivity to decrease.

At the system level, the simulation of the IsOWC system implementing the homodyne BPSK modulation technique has been reported. The magnitude degradation of the lasers and photodiodes are fed to the IsOWC system and simulated. The impact of radiation can be seen along with the degradation of the system performance with radiation doses. The combined effect of irradiation on both Tx and Rx at the performance level has been modeled analytically and by simulation. After the 4^th^ dose of the irradiation process, the system performance degraded by 70% in electron approximately, where the Q factor decreases by more than 80% approximately and the system BER increase by more than 20 orders of magnitude and reach 1 × 10^−3^; which indicates that further irradiation processes were not necessary. This fact has been translated in the analytical and simulation modeling, where the combined radiation effect on the Tx and Rx has been reported and compared with state of art investigations.
